# Albumin-Bilirubin Grade, Tumor Characteristics, and Survival of Patients with Hepatocellular Carcinoma

**DOI:** 10.1159/ocl/aebag006

**Published:** 2026-08-27

**Authors:** Rossella Donghia R, Brian I. Carr

**Affiliations:** a Data Science Unit, National Institute of Gastroenterology - IRCCS “Saverio de Bellis”, Bari, Italy; b Liver Transplant Institute, İnönü University, Malatya, Turkey

**Keywords:** Hepatocellular carcinoma, Albumin-bilirubin, Portal vein thrombosis, Maximum tumor diameter, Survival

## Abstract

**Introduction:**

Albumin-bilirubin (ALBI) grade has recently been introduced as an objective measure of liver function and has been shown to be useful in prognostication for patients with hepatocellular carcinoma (HCC). The reasons for this usefulness in HCC prognostication are not clear.

**Methods:**

The study aimed to determine, in HCC patients, whether ALBI grade correlates with indices of HCC aggressiveness. A large cohort of nonsurgical HCC patients was examined for ALBI grade in relation to maximum tumor diameter (MTD), tumor focality, percentage of patients with portal vein thrombosis (PVT), serum alpha-fetoprotein (AFP) levels and survival.

**Results:**

ALBI grade was significantly associated with HCC aggressiveness parameters, particularly AFP, PVT, and tumor multifocality, but not with MTD. Similar associations were observed in the subset of patients with low AFP levels. ALBI grade showed a strong and graded relationship with survival, comparable to that observed for serum albumin alone. In Cox models stratified by ALBI categories, AFP, PVT, and tumor multifocality were independently associated with mortality.

**Conclusions:**

These findings suggest that ALBI reflects not only liver function, but also correlates with tumor aggressiveness features.

## Plain Language Summary

Alpha-fetoprotein (AFP) is the most frequently used blood biomarker in patients with hepatocellular carcinoma (HCC), but is not elevated in more than half of patients and even then, is unhelpful in patients with small tumors. ALBI (albumin-bilirubin) has gained popularity in recent years as a quantitative estimate for liver function and also as an HCC prognostic marker. However, its mechanism is generally unclear, and its use in low AFP patients has not been determined. In this paper, we explore both of these knowledge gaps and show that ALBI grade relates significantly to multifocality and portal vein thrombosis, but not to HCC size, in patients with both elevated and normal blood AFP levels.

## Introduction

The albumin-bilirubin (ALBI) grade is derived from a validated nomogram to objectively assess prognosis and liver function in patients with hepatocellular carcinoma (HCC) [1]. It is based solely on serum albumin and bilirubin levels, thus excluding evaluation of subjective variables such as ascites and encephalopathy and categorizes patients into 3 prognostic risk categories [2]. Recent evidence suggests ALBI grading at baseline can predict prognosis in HCC patients [3, 4]. ALBI grade was initially calculated by a predictor [2], but was more recently simplified by an automatic calculator, using MDCalc (www.mdcalc.com), based on ALBI formula= (log10 (total bilirubin [mg/dL] × 17.1) × 0.66) + (albumin [g/dL] × 10 × −0.085) [5]. The scores are defined as grade 1 (≤−2.60), grade 2 (more than −2.60 to ≤ −1.39), and grade 3 (more than −1.39). Grade 1 has the best prognosis and grade 3 has the worst prognosis. ALBI has been shown to be useful for HCC patients treated with radiotherapy [6], immune checkpoint inhibitor immunotherapies [7, 8], chemoembolization [9, 10], radioembolization [11, 12], resection [13], liver transplantation both within and beyond Milan [14–16], and in resected low alpha-fetoprotein (AFP) patients [17]. ALBI provides similar prognostication as for inflammation-based indices [18], and its predictive ability has been reported to be enhanced by the addition of serum gamma-glutamyl transferases (GGTs) [19]. It has also proven useful across Barcelona Clinic Liver Cancer (BCLC) stages [20].

However, the reasons for ALBI prognostic ability are not so clear. Is it useful just because it reflects liver function, as developed, or does ALBI also somehow reflect or even influence HCC aggressiveness indices, such as AFP, tumor size or maximum tumor diameter (MTD), tumor focality, or portal vein thrombosis (PVT)? This study aimed to examine how ALBI grades correlate with these tumor parameters, as a first step to answer these questions.

## Methods

### Clinical Database

A clinical HCC database with 6,759 patients that we have previously interrogated [21] was further examined. The data were collected from the Italian Liver Cancer (ITA.LI.CA) database. Patients had routine hematology, liver function tests, Child-Pugh score constituents (albumin, total bilirubin, prothrombin time, and presence/absence of ascites and encephalopathy) and AFP levels recorded on their initial clinic visit, as well as baseline tumor characteristics of MTD, tumor number, and presence/absence of macroscopic malignant PVT by Computed Axial Tomography scan evaluation. ALBI levels were calculated using MDCalc (www.mdcalc.com). All patients were diagnosed according to European Association for the Study of Liver Disease (EASL) criteria. Macroscopic PVT by HCC was assessed by the presence of heterogeneous contrast-enhanced PVT with enlargement (>23 mm) in proximity to vascular HCC.

### Statistical Analysis

Patients’ parameters are reported as median and interquartile range for continuous variables, and as frequency and percentage (%) for categorical variables. Comparisons between independent groups (ALBI categories) were performed with Kruskal-Wallis test for a number of groups of 3 for continuous variables, while for categorical variables, chi-square was used. The associations between single parameters and risk of mortality were tested with a Cox model, with hazard ratio (HR) as the estimate with confidence interval of 95% (CI 95%) stratified by ALBI categories.

The performance of the Cox proportional hazard models was assessed through the concordance index (C-index), the Akaike Information Criterion (AIC), and the Bayesian Information Criterion (BIC). The C-index quantified the model’s discriminative capacity, reflecting its ability to correctly rank individuals according to their risk of experiencing the event. In contrast, AIC and BIC were employed to compare model fit, with lower values denoting a more parsimonious and better-fitting model by accounting for both model accuracy and complexity.

Survival probability on categorized liver parameters was explored using a nonparametric Kaplan-Meier method. The equality of survival curves was analyzed using the log-rank test. To test the null hypothesis of non-association, the two-tailed probability level was set at 0.05. Analyses were conducted with Stata Statistical Software: Release 18, StataCorp, 2023, StataCorp LLC. (College Station, TX, USA).

### Ethical Considerations

The data was collected from the Italian Liver Cancer (ITA.LI.CA) database of HCC patients. ITA.LI.CA database management conforms to Italian legislation on privacy and this study conforms to the ethical guidelines of the Declaration of Helsinki. The study design was approved (protocol n. 99/2012/O/Oss) by the Independent Ethic Committee of S. Orsola-Malpighi hospital of Bologna, which operates as coordinating center of the ITA.LI.CA network. Inonu University Ethics committee approval was 2022/3905 for retrospective analysis of de-identified and deceased HCC patients. Inonu University Ethics committee considered that informed consent was not required for this study as the patients were deceased at the time of this retrospective study and the study was based on publicly available data.

## Results

Comparison between parameters across ALBI groups in patients with MTD of ≤6 cm. Tumor (AFP, PVT, focality, and MTD) and blood parameters are reported in Table 1. Variables are presented as both continuous and categorical, with clinical cut-off. All variables showed statistical differences across ALBI groups with a *p* value of <0.05, with the exception of GGT as categorical (*p* = 0.35) and MTD (*p* = 0.63 and *p* = 0.49, respectively). AFP, multifocality and presence of PVT significantly increased with an increase in ALBI grade and conversely, serum albumin levels and Karnofsky index decreased with an increase in ALBI grade. Locoregional therapies were given across ALBI grades, but resection was given predominantly to patients with ALBI grade 1. The predominant ALBI grade for palliative care patients was 3. The distribution of patients was similar for patients with HBV and hepatitis C, with the largest group of patients being in grade 1. However, patients with alcoholic etiology had a gradual increase in the percentage of patients as ALBI grade increased. The same approach was used to analyze these parameters in the subcohort of patients with low serum AFP levels and showed similar results (online suppl. Table 1; for all online suppl. material, see https://doi.org/10.1159/ocl/aebag006).

**Table 1.** Comparison between parameters in ALBI groups in patients with MTD of ≤6 cm (*n* = 5,821)

Parameters^a^	ALBI			*p* value
	grade 1 (*n* = 1,149) (22.35%)	grade 2 (*n* = 3,348) (65.12%)	grade 3 (*n* = 644) (12.53%)	
Gender (M) (%)	910 (79.20)	2,484 (74.19)	492 (76.40)	0.003
Total bilirubin (mg/dL)	0.70 (0.50)	1.20 (0.80)	3.00 (3.10)	0.0001^c^
Total bilirubin (mg/dL)				<0.001
≤1.50	1,094 (95.21)	2,350 (70.19)	101 (15.68)	
>1.50	55 (4.79)	998 (29.81)	543 (84.32)	
Albumin (g/dL)	4.20 (0.40)	3.50 (0.50)	2.60 (0.40)	0.0001^c^
Albumin (g/dL)				<0.001
≤3.50	2 (0.17)	1,855 (55.41)	642 (99.69)	
>3.50	1,147 (99.83)	1,493 (44.59)	2 (0.31)	
Platelets (×10^9^/L)	144.00 (90.00)	103.00 (77.00)	89.00 (68.50)	0.0001^c^
Platelets (×10^9^/L)				<0.001
≤100	290 (26.01)	1,516 (47.46)	363 (58.55)	
>100	825 (73.99)	1,678 (52.54)	257 (41.45)	
AFP (IU/mL)	8.48 (36.00)	14.00 (69.00)	21.80 (233.80)	0.0001^c^
AFP (IU/mL)				<0.001
≤100	853 (83.71)	2,313 (77.96)	390 (68.54)	
>100	166 (16.29)	654 (22.04)	179 (31.46)	
GGT (IU/mL)	1.50 (2.00)	1.90 (2.57)	1.86 (2.81)	0.0001^c^
GGT (IU/mL)				0.35
≤100.00	1,023 (97.34)	2,905 (97.22)	567 (98.27)	
>100.00	28 (2.66)	83 (2.78)	10 (1.73)	
Hemoglobin (g/dL)	13.90 (2.30)	12.90 (2.60)	11.80 (2.60)	0.0001^c^
Hemoglobin (g/dL)				<0.001
≤12.20	217 (20.49)	1,128 (38.51)	344 (59.62)	
>12.20	842 (79.51)	1,801 (61.49)	233 (40.38)	
Leukocytes (×10^9^/L)	5,090.00 (3,530.00)	4,260.00 (3,460.00)	4,100.00 (6,184.85)	0.0001^c^
Leukocytes (×10^9^/L)				<0.001
≤4,000.00	298 (34.33)	971 (45.95)	171 (48.58)	
>4,000.00	570 (65.67)	1,142 (54.05)	181 (51.42)	
Lymphocytes (×10^9^/L)	1,555.00 (910.00)	1,255.00 (790.00)	1,215.00 (1,030.00)	0.0001^c^
Lymphocytes (×10^9^/L)				<0.001
≤1,200.00	102 (31.48)	336 (47.19)	53 (50.00)	
>1,200.00	222 (68.52)	376 (52.81)	53 (50.00)	
MTD (cm)	2.50 (1.70)	2.50 (1.90)	2.60 (2.20)	0.63^c^
MTD (cm)				0.49
<3	673 (58.57)	1,907 (56.96)	360 (55.90)	
3–6	476 (41.43)	1,441 (43.04)	284 (44.10)	
PVT (yes)	73 (6.43)	458 (13.83)	169 (26.61)	<0.001
Focality (>1)	260 (23.38)	949 (29.39)	199 (32.62)	<0.001
Karnofsky Index	100.00 (10.00)	90.00 (10.00)	80.00 (30.00)	0.0001
Treatment (%)				<0.001
TACE/TAE/TARE	229 (21.07)	878 (28.99)	118 (23.46)	
Resection	305 (28.06)	422 (13.93)	17 (3.38)	
Palliative care	22 (2.02)	157 (5.18)	146 (29.03)	
Aetiology (%)				<0.001
HBV	133 (12.41)	248 (7.88)	44 7.18)	
HVC	549 (51.21)	1,610 (51.18)	261 (42.58)	
Alcohol	132 (12.31)	485 (15.42)	122 (19.90)	

AFP, alpha-fetoprotein; GGT, gamma-glutamyl transpeptidase; MTD, maximum tumor diameter; PVT, portal vein thrombosis; TACE, trans-arterial chemoembolization; TAE, trans-arterial embolization; TARE, radioembolization; HBV, hepatitis B; HCV, hepatitis C.

^a^ As median and interquartile range (IQR) for continuous variables, and as frequency and percentage (%) for categorical variables.

^b^ Chi-square test.

^c^ Kruskal-Wallis rank test.

### Multinomial Logistic Regression Analysis

A multinomial logistic regression analysis was performed to evaluate the association between ALBI categories and single parameters in the models (Table 2). Platelets, hemoglobin, leukocytes, and lymphocytes had a protective role, with relative risk reduction of <1 in ALBI categories in a statistically significant manner. In contrast, with AFP >100 IU/mL, presence of PVT or multifocality, the risk increased (relative risk reduction of >1) in ALBI grades 2 and 3 compared with grade 1.

**Table 2.** Multinomial logistic regression model of ALBI categories (grade 1 as reference category) on single variables in patients with MTD of ≤6 cm

Parameters	Grade 2				Grade 3			
	RRR	se (RRR)	*p* value	95% CI	RRR	se (RRR)	*p* value	95% CI
Platelets (×10^9^/L)	0.99	0.001	<0.001	0.99–1.00	0.99	0.001	<0.001	0.99–1.00
Platelets (×10^9^/L)								
≤100	–	–	–	–	–	–	–	–
>100	0.39	0.03	<0.001	0.33–0.45	0.25	0.03	<0.001	0.20–0.31
AFP (IU/mL)	1.00	4.36e−06	0.65	0.99–1.00	1.00	4.99e−06	0.14	0.99–1.00
AFP (IU/mL)								
≤100	–	–	–	–	–	–	–	–
>100	1.45	0.14	<0.001	1.20–1.75	2.36	0.29	<0.001	1.85–3.01
GGT (IU/mL)	0.99	0.001	0.70	0.99–1.00	0.99	0.01	0.06	0.99–1.00
GGT (IU/mL)								
≤100.00	–	–	–	–	–	–	–	–
>100.00	1.04	0.23	0.85	0.68–1.61	0.64	0.24	0.24	0.31–1.34
Hemoglobin (g/dL)	0.75	0.01	<0.001	0.72–0.78	0.58	0.02	<0.001	0.55–0.62
Hemoglobin (g/dL)								
≤12.20	–	–	–	–	–	–	–	–
>12.20	0.41	0.03	<0.001	0.35–0.49	0.17	0.02	<0.001	0.14–0.22
Leukocytes (×10^9^/L)	0.99	0.001	<0.001	0.99–1.00	0.99	0.01	0.007	0.99–1.00
Leukocytes (×10^9^/L)								
≤4,000.00	–	–	–	–	–	–	–	–
>4,000.00	0.61	0.05	<0.001	0.52–0.72	0.55	0.07	<0.001	0.43–0.71
Lymphocytes (×10^9^/L)	0.99	0.001	<0.001	0.99–1.00	0.99	0.01	0.008	0.99–1.00
Lymphocytes (×10^9^/L)								
≤1,200.00	–	–	–	–	–	–	–	–
>1,200.00	0.51	0.07	<0.001	0.39–0.68	0.46	0.10	0.001	0.29–0.72
MTD (cm)								
<3	–	–	–	–	–	–	–	–
3–6	1.07	0.07	0.35	0.93–1.22	1.11	0.11	0.27	0.92–1.35
PVT (yes)	2.34	0.31	<0.001	1.81–3.02	5.28	0.79	<0.001	3.93–7.09
Focality (>1)	1.36	0.11	<0.001	1.16–1.60	1.59	0.18	<0.001	1.27–1.97

RRR, relative risk ratio; se (RRR), standard error of RRR; AFP, alpha-fetoprotein; GGT, gamma-glutamyl transpeptidase; MTD, maximum tumor diameter; PVT, portal vein thrombosis.

### ALBI in Relation to Survival

A Cox model on mortality on single variables inserted in the model is shown in Table 3. AFP levels as both continuous and categorical variables were statistically significant with HR >1.0 at risk for death, *p* < 0.05. Patients with ALBI grade 2 showed a significantly increased risk (HR = 1.42, 1.27–1.59, 95% CI, *p* < 0.001). The risk was even higher in ALBI grade 3, with a nearly threefold increase compared with grade 1 as reference category (HR = 2.74, 2.36–3.18, 95% CI, *p* < 0.001). In the MTD category (3–6 cm), patients were at risk for mortality in ALBI grades 2 and 3 (HR >1, *p* < 0.05). Patients having PVT or tumor multifocality represented a high risk (HR) for death in all 3 ALBI categories, whereas hemoglobin, leucocytes and lymphocytes were protective (HR <1). Mortality was associated with high platelet levels only in ALBI grade 3 (HR = 1.20, *p* = 0.04, 1.01–1.44, 95% CI).

**Table 3.** Cox proportional hazard models for death on clinical parameters in HCC patients with MTD of ≤6 cm, stratified by ALBI categories

Parameters	ALBI											
	grade 1				grade 2				grade 3			
	HR	se (HR)	*p* value	95% CI	HR	se (HR)	*p* value	95% CI	HR	se (HR)	*p* value	95% CI
Platelets (×10^9^/L)	0.99	0.01	0.28	0.99–1.00	1.00	0.001	0.04	1.00–1.01	1.00	0.001	0.54	0.99–1.00
Platelets (×10^9^/L)												
≤100 [Ref.]	–	–	–	–	–	–	–	–	–	–	–	–
>100	0.97	0.09	0.75	0.80–1.17	0.99	0.04	0.79	0.91–1.08	1.20	0.11	0.04	1.01–1.44
AFP (IU/mL)	1.00	3.26e−06	0.001	1.00–1.01	1.00	1.51e−06	<0.001	1.00–1.01	1.00	3.06e−06	0.02	1.00–1.01
AFP (IU/mL)												
≤100 [Ref.]	–	–	–	–	–	–	–	–	–	–	–	–
>100	1.60	0.17	<0.001	1.30–1.97	1.72	0.09	<0.001	1.56–1.90	1.53	0.15	<0.001	1.27–1.86
GGT (IU/mL)	1.00	0.01	<0.001	1.00–1.01	1.001	0.001	0.02	1.00–1.01	1.00	0.01	0.23	0.99–1.01
GGT (IU/mL)												
≤100.00 [Ref.]	–	–	–	–	–	–	–	–	–	–	–	–
>100.00	2.12	0.87	0.07	0.95–4.74	1.81	0.31	0.001	1.29–2.52	1.25	0.51	0.59	0.56–2.79
Hemoglobin (g/dL)	0.95	0.02	0.08	0.91–1.00	0.91	0.01	<0.001	0.89–0.93	0.95	0.02	0.05	0.91–1.00
Hemoglobin (g/dL)												
≤12.20 [Ref.]	–	–	–	–	–	–	–	–	–	–	–	–
>12.20	0.78	0.09	0.03	0.63–0.97	0.71	0.03	<0.001	0.65–0.78	0.83	0.08	0.05	0.69–0.99
Leukocytes (×10^9^/L)	1.00	0.01	0.70	0.99–1.00	1.00	9.71e−06	0.72	0.99–7.00	0.99	0.001	0.32	0.99–1.00
Leukocytes (×10^9^/L)												
≤4,000.00 [Ref.]	–	–	–	–	–	–	–	–	–	–	–	–
>4,000.00	0.98	0.11	0.87	0.79–1.22	0.96	0.05	0.44	0.85–1.07	0.94	0.12	0.61	0.73–1.20
Lymphocytes (×10^9^/L)	0.99	0.01	0.76	0.99–1.00	0.99	0.001	0.55	0.99–1.00	1.00	0.01	0.82	0.99–1.00
Lymphocytes (×10^9^/L)												
≤1,200.00 [Ref.]	–	–	–	–	–	–	–	–	–	–	–	–
>1,200.00	0.96	0.21	0.86	0.62–1.49	0.83	0.09	0.10	0.67–1.04	0.96	0.25	0.88	0.59–1.57
MTD (cm)	1.05	0.03	0.12	0.99–1.11	1.05	0.02	0.002	1.02–1.08	1.09	0.03	0.005	1.06–1.16
MTD (cm)												
<3 [Ref.]	–	–	–	–	–	–	–	–	–	–	–	–
3–6	1.12	0.09	0.18	0.95–1.32	1.18	0.05	<0.001	1.09–1.28	1.32	0.12	0.001	1.11–1.57
PVT (Yes)	2.63	0.40	<0.001	1.95–3.54	2.59	0.15	<0.001	2.31–2.90	2.22	0.22	<0.001	1.82–2.70
Focality (>1)	1.58	0.15	<0.001	1.31–1.91	1.31	0.06	<0.001	1.19–1.43	1.14	0.11	0.16	0.95–1.38

HR, hazard ratio; se (HR), standard error of HR; AFP, alpha-fetoprotein; GGT, gamma-glutamyl transpeptidase; MTD, maximum tumor diameter; PVT, portal vein thrombosis.

The same analyses were performed for combined variables in models adjusted for covariates. No statistically significant association was observed for the combination variables. Performance was calculated as C-index, AIC, and BIC. Model fit indices were as follows: for the ALBI model, AIC = 30,162.08 and BIC = 30,202.19; and for the combination model, AIC = 784.73 and BIC = 800.77 (online suppl. Table 2).

Survival probability was also evaluated with the Kaplan-Meier method and relative log-rank test to evaluate the differences between curves, as shown in Figure 1. Each parameter showed significant survival differences between its laboratory values. Of note, the results for ALBI and for serum albumin levels in patients with normal serum total bilirubin levels were essentially identical. A similar approach was taken for treatments, as shown in online supplementary Figure 1a, with best survival being for locoregional therapies and worst survival for palliative care patients. Survival was similar across aetiologies, as shown in online supplementary Figure 1b.

**Fig. 1.**
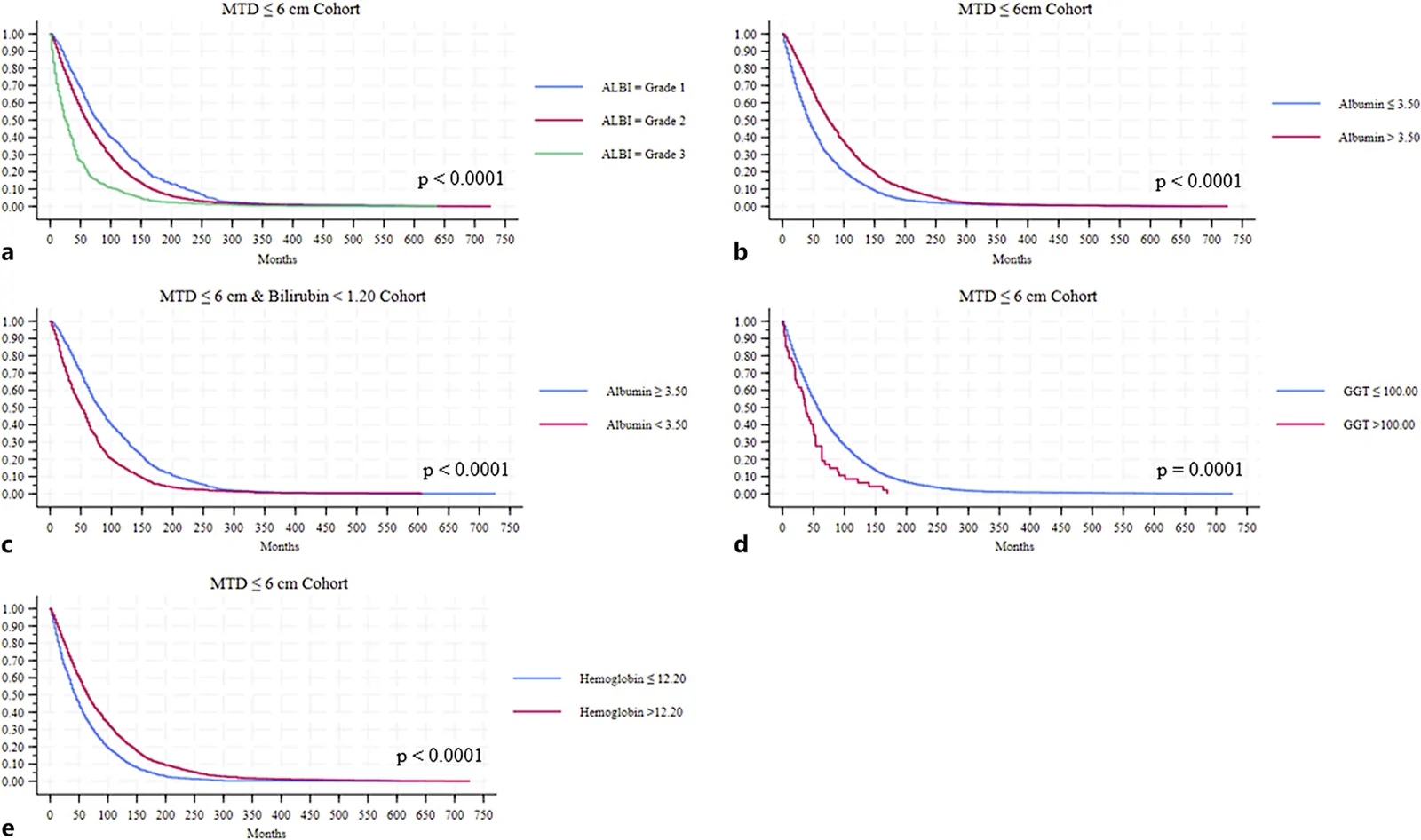
Kaplan-Meier plots of survival for ALBI grades (**a**); albumin, g/dL (**b**); albumin in low bilirubin (<1.2 mg/dL) sub-cohort (**c**); GGT, IU/mL (**d**); and hemoglobin, g/dL (**e**).

## Discussion

Many classification and prognostication scores for HCC have been published, on single parameters reflecting HCC biomarkers (AFP, DCP), on liver function tests (albumin, bilirubin, alkaline phosphatase or inflammation markers (NLR, PLR, CRP, albumin, and Glasgow score combination of C-reactive protein and albumin levels), or on combinations of parameters that include both liver and tumor parameters (Okuda; CLIP, Carcinoma of the Liver Italian program; BCLC, Barcelona Clinic Liver cancer; JIS, Japan Integrated Staging) [22–27]. The great variety of these scores reflects both the heterogeneity of HCC phenotypes as well as the large variety of both tumor and non-tumor factors that relate to HCC prognosis. One of the most extensively studied and important prognostic factors is the serum level of AFP, yet more than 70 years after its significance was published [28, 29], there is still no agreement on the explanation for its prognostic power or actions in HCC biology [30]. ALBI, based upon serum levels of albumin and total bilirubin, was recently introduced into clinical practice as a quantitative measure of liver function and for its usefulness in HCC prognostication [1, 2, 4]. The usefulness of ALBI has been validated in relation to most HCC treatment modalities [6–16] and has also been shown to be prognostically useful in the current report, even in the large subset of HCC patients who do not have elevated serum AFP levels (online suppl. Table 1). However, the reasons underlying the prognostic usefulness of both albumin and ALBI have not been clearly identified. ALBI score is a continuous variable and serves as an objective marker of liver function in cirrhosis or HCC, even in the presence of normal bilirubin values of <1.2 mg/dL.

Although ALBI and its constituent albumin are prognostic factors for HCC, it is not quite clear how or why, beyond the idea that they reflect the liver damage that occurs as the HCC grows. In the current work, we show that ALBI appears to be more than just an index of liver function, as it is independently associated with PVT, multifocality and elevated AFP, but not with tumor size. It could thus reflect the tumor microenvironment, systemic inflammatory status, liver/tumor bidirectional interaction, or more aggressive tumor biology. Changes in serum albumin levels have also been shown to decrease with tumor cachexia [31] as a marker of cancer-associated inflammation [27, 32] and with HCC aggressiveness [33]. Conversely, experimental albumin manipulation has been shown to decrease HCC growth [34–36]. In fact, it has been suggested that the prognostic power of ALBI relates mainly to its albumin component [11].

It is interesting that ALBI related significantly to PVT and multifocality, but not to MTD. PVT and multifocality share the property of tumor cell migration, as opposed to MTD, which reflects mainly tumor growth. Inflammatory indices and hypoalbuminemia seem to be related to PVT and multifocality [21, 37]. While HCC growth is also related to inflammation, multiple cytokine, chemokine, and multiple growth factor pathways [38]. Furthermore, large-size HCCs have been shown to be associated both with and without inflammation [39–43], suggesting both an inflammation-related and an inflammation-unrelated set of mechanisms. Furthermore, non-cirrhotic HCCs may have larger HCCs and better survival as they are more often unifocal and can be resected [40]. Non-cirrhotic HCC patients also tended to have higher serum albumin levels and better overall survival [41]. Thus, larger HCC size may reflect the actions of factors other than inflammation, or at least more than one pathway to HCC growth. Although the association and predilection of HCC for cirrhosis have been known for many years, the mechanisms involved in cirrhosis being a precursor for HCC still remain to be clarified. Their association would seem to be bidirectional, as a growing mass in the liver can displace or destroy non-tumor liver parenchyma; but contrariwise, an HCC is subject to the many microenvironmental influences of its surrounding (and inflamed) liver. In this regard, recent reports of effective antiviral therapy in HCC patients support the idea that the treatment of the inflamed hepatic microenvironment may alter the HCC natural history [44–46], even though HCC recurrence after sustained virological responses still occurs [47]. Even the non-anti-viral, anti-inflammatory drug aspirin appears to improve survival in patients with advanced HCC compared with tyrosine kinase inhibitors alone [48]. In addition, HCC radioembolization treatment seems able to improve underlying liver function in some patients [49, 50].

Although the underlying biological mechanisms warrant further investigation, our results clearly demonstrate the prognostic significance of both serum albumin and ALBI grade and the significant correlation of ALBI with PVT and tumor focality. Thus, there is an association, but causative mechanisms are currently only hypothesized.

Strengths of this paper are the demonstration of the correlation of both PVT and tumor focality with ALBI, including in patients with either normal or elevated AFP levels, as a likely explanation of its prognostic power. Weaknesses include both the retrospective nature of the analysis and the absence of clear mechanisms.

## Statement of Ethics

The data were collected from the Italian Liver Cancer (ITA.LI.CA) database of HCC patients. ITA.LI.CA database management conforms to Italian legislation on privacy, and this study conforms to the ethical guidelines of the Declaration of Helsinki. The study design was approved (protocol n. 99/2012/O/Oss) by the Independent Ethics Committee of S. Orsola-Malpighi hospital of Bologna, which operates as coordinating center of the ITA.LI.CA network. Inonu University Ethics Committee approval was 2022/3905 for retrospective analysis of de-identified and deceased HCC patients. Inonu University Ethics Committee considered that informed consent was not required for this study as the patients were deceased at the time of this retrospective study and the study was based on publicly available data.

## Conflict of Interest Statement

The authors declare no conflict of interest. All authors read and agree with the contents of this paper, which was not previously published.

## Funding Sources

This work was supported in part by NIH grant CA 82723 (BIC) and by the Italian Ministry of Health, Ricerca Corrente 2025–2026. These funds were used for salary support for the 2 investigators for this study.

## Author Contributions

R.D.: statistics and writing. B.I.C.: concept and writing.

## Data Availability

The data that support the findings of this study are not publicly available due to the patient data not being public, i.e. their containing information that could compromise the privacy of research participants, but are available from the authors (R.D. and B.I.C.) on reasonable request. E-mails: rossella.donghia@irccsdebellis.it; brianicarr@hotmail.com.
